# Tobacco smoking and biomarker profile among beverage industrial workers and their spouses in Rwanda: A cross-sectional study

**DOI:** 10.1371/journal.pgph.0003946

**Published:** 2025-07-03

**Authors:** Charles Nsanzabera, Jean claude Rukundo, Mustafe Yusuf Said, Leonard Ndayisenga

**Affiliations:** 1 Department of Public health, African Institute of Research for Public Health and Development, Kigali, Rwanda; 2 Department of Public Health, Jomo Kenyatta University of Agriculture and Technology, Nairobi, Kenya; 3 Public health department, Mount Kenya University Kigali Campus, Kigali, Rwanda; 4 Department of Public health, University of Hargeisa college of medicine and health sciences, Hargeisa, Somaliland; 5 Department of Occupational Health and Safety service, University Teaching Hospital of Butare, Huye, Rwanda; PLOS: Public Library of Science, UNITED STATES OF AMERICA

## Abstract

Globally, smoking leads to over 7 million preventable deaths annually, with higher rates in men (16%) than women (7%). Although smoking rates have declined in high-income countries, tobacco use in Rwanda remains relatively low, with 14% of men and 3% of women affected. The focus on beverage industry workers and their spouses is justified by their higher income levels and potential exposure to stressful occupational factors that are commonly associated with smoking. The study aimed to assess tobacco smoking prevalence and the biomarker profiles of beverage industrial workers and their spouses in Rwanda. This research employed a cross-sectional study design conducted in a beverage manufacturing industry in Rwanda. The study targeted 822 individuals, including beverage industry employees and their spouses, aged 30–75 years. An initial sample size of 384 was calculated using the Cochrane formula, which was adjusted to 440 to account for non-responses. Data collection was conducted from May to December 2018. Data analysis was conducted using SPSS version 22, with chi-square and logistic regression tests to assess tobacco use and other associated factors with a significance cutoff of p < 0.05 at 95% CI. The study controlled for confounders by increasing the sample size and using stratified and simple random sampling to ensure representativeness. Additionally, the multicollinearity test with VIF and selection of variables for multivariate analysis were ensured. The analysis reveals 6.8% were smokers and several key predictors of smoking behavior. Participants with elevated cardiovascular disease (CVD) risk (≥10%) have significantly higher odds of smoking, with an adjusted odds ratio of 2.95 (95% CI: 1.10-7.88), suggesting that CVD risk is a crucial factor in smoking behavior due to overlapping lifestyle risks. Additionally, high serum uric acid (SUA) levels (≥7 mg/dl) are strongly associated with smoking, with an adjusted odds ratio of 4.28 (95% CI: 1.14-11.87), indicating that elevated SUA levels are over four times more likely to be linked to smoking. Age is another significant predictor, with participants aged 50 years or older being nearly three times more likely to smoke compared to younger individuals, as shown by an adjusted odds ratio of 2.77 (95% CI: 1.13-6.80). Participants with hypertension or those treated for hypertension have lower adjusted odds ratio of 0.38 (95% CI: 0.10-1.45). The study’s findings could guide public health policymakers in designing workplace-based smoking cessation programs tailored to industry employees and their spouses. The study found that tobacco smoking is relatively low in this population, with elevated cardiovascular disease risk. Also, there are significant associations between tobacco smoking and elevated CVD risk, high SUA levels, and older age. Policymakers should implement targeted awareness campaigns and education programs addressing the specific risk factors identified.

## Introduction

Globally, smoking stands as the leading preventable cause of death, claiming over 7 million lives annually and accounting for 12% of deaths among adults aged 30 and above [1]. Alarming projections suggest this toll will rise to 8 million deaths annually in the coming decades, underscoring the urgent need to understand its biological consequences. Biomarkers provide invaluable insights into the impact of smoking, serving as measurable indicators of the body’s response to harmful substances like nicotine, carbon monoxide, and carcinogenic chemicals [2,3]. Smoking profoundly alters biomarker levels, with elevated cotinine reflecting nicotine exposure, heightened malondialdehyde indicating oxidative stress, and increased C-reactive protein signaling systemic inflammation [4,5]. These shifts highlight the cellular damage, inflammation, and cardiovascular strain associated with tobacco use [6].

Historical trends show that smoking was most prevalent in high-income Western countries, but significant reductions have been achieved through public health interventions [2,3]. In contrast, tobacco use has increased in several Middle Eastern and African countries, including those in the East African Community (EAC), where smoking-related fatalities rank among the top five causes of death [6].

In East Africa, smoking prevalence varies significantly across countries [7].Rwanda, according to the 2015 Demographic Health Survey, has a relatively low smoking prevalence compared to its neighbors. However, within the population, men (14%) exhibit a far higher smoking rate than women (3%) [8]. Despite these lower rates, the associated health risks, including cardiovascular disease (CVD), remain substantial. This underscores the need for localized research to better understand smoking behaviors and related health outcomes within Rwanda’s context [9].

Tobacco smoking is a well-documented risk factor for various cardiovascular diseases, including coronary artery disease, peripheral arterial disease, and stroke. Smoking contributes to atherosclerosis through mechanisms such as vascular damage, lipid peroxidation, and thrombosis [10]. Globally, approximately 5.9 million premature deaths from CVD in 2013 were attributed to smoking [11]. Furthermore, research links smoking with dyslipidemia, elevating triglycerides, total cholesterol, and LDL cholesterol while lowering HDL cholesterol, compounding its detrimental effects on cardiovascular health [12,13].

Biomarkers, such as serum uric acid and lipid profiles, play a crucial role in assessing the impact of smoking on health [14]. Smoking has been associated with abnormal lipid metabolism, which exacerbates cardiovascular risk [15]. However, research presents mixed findings regarding its direct role in hypertension, necessitating further investigation. Nicotine and carbon monoxide in tobacco smoke contribute to endothelial dysfunction and impaired vascular function, making biomarkers invaluable in identifying early signs of smoking-related damage and guiding preventive measures [16].

While numerous studies have explored the link between smoking and CVD, gaps remain regarding occupational and lifestyle factors that influence smoking behaviors, particularly in Rwanda [17]. Beverage industrial workers may face occupational stressors that promote smoking as a coping mechanism, while their spouses offer insights into household and social influences [18]. Previous studies have largely focused on general populations, leaving a gap in understanding these specific dynamics and their implications for biomarker profiles [19].

Beverage industrial workers and their spouses represent a distinctive population that may face specific risks related to smoking and its health consequences. Exploring the occupational factors associated with smoking behaviors and identifying biomarkers linked to cardiovascular disease risk are essential steps toward addressing these risks [20]. The focus on beverage industry workers and their spouses is justified by their higher income levels and potential exposure to stressful occupational factors that are commonly associated with smoking [21]. The findings from such research can guide targeted interventions to reduce smoking rates and improve cardiovascular health, offering valuable insights for policymakers, healthcare providers, and public health initiatives. Therefore, this study aims to bridge existing knowledge gaps by investigating tobacco smoking and its association with biomarker profiles among this unique population in Rwanda.

## Methods and materials

### Study design and sampling

This research utilized a cross-sectional study design conducted from May to December 2018 within a beverage manufacturing industry setting in Rwanda. The study participants included both industry employees and their spouses to gather information from both the workplace and the community. The target population consisted of 822 individuals, with participants ranging in age from 30 to 75 years. The inclusion criteria required participants to be either employees of the industry or their spouses, and to be within the specified age range from 30 to 75 years. The age range selection of 30–75 was used to meet the requirements of cardiovascular diseases prediction with cox regression and minimize lower age and higher confounders. According to the World Health Organization (WHO) categorization, the age group extends up to 75 years, which justified its inclusion in the study design. However, it is important to note that while the study set an upper age limit of 75 years, no participants in the study sample actually reached that age. This approach ensured methodological consistency while aligning with global health standards for age classification in epidemiological research. Therefore, this helped to control the confounder related to higher age that tobacco use is linked to high risk of cardiovascular disease risk while it is due to the higher age. Individuals with clinically diagnosed cardiovascular disease were excluded to prevent bias in assessing CVD risk. Sample size calculation used the Cochrane formula, resulting in an initial sample size of 384 [22]. Adjusting for a 12% non-response rate, the corrected sample size was calculated as 437.5, rounded to 440. Of the 440 participants, 270 were employees and 170 were spouses. The study population was divided into four strata: Kicukiro plant workers, Kicukiro plant workers’ spouses, Rubavu plant workers, and Rubavu plant workers’ spouses. This stratification ensured that each subgroup was appropriately represented in the study. To achieve a representative sample, a combination of sampling techniques was employed. Stratified random sampling was first used to divide the population into these distinct strata, allowing for the selection of participants from each subgroup based on their proportional size within the population. Following this, simple random sampling was applied within each stratum to randomly select individual participants, ensuring that every eligible member within a stratum had an equal chance of being included. This combined approach enhanced the representativeness and generalizability of the study findings.

### The dependent and independent variable of this study

The tobacco products use was the dependent variable of this study. The age, gender, employment status and body mass index were taken as the independent’s variables. Additionally, blood lipid, diabetes, hypertension, and cardiovascular diseases risk predicted by Framingham general risk score were also cross tabulated to measure its association with tobacco use. The cardiovascular disease risk was categorized as elevated risk (>=10%) and low risk (<10%) [23]. The blood lipids were measured according NCEP (National Cholesterol Education Program) and ATPIII (Adult Treatment Panel). Low-Density Lipoprotein (LDL) levels were categorized as follows: borderline high at 130–159 mg/dl, high at 160–189 mg/dl, and very high at ≥190 mg/dl. Dyslipidaemia was defined by a total cholesterol level above 270 mg/dl [23]. According to the NCEP ATP III guidelines, triglyceride levels were categorized as follows: < 150 mg/dl was considered normal, 150–199 mg/dl was borderline high, 200–499 mg/dl was high, and ≥500 mg/dl was very high. Serum uric acid levels were categorized as normal if <7 mg/dl and high if >7 mg/dl [24].

### Study instruments

The study utilized an instrument composed of three sections: a standardized questionnaire covering socio-demographic information and tobacco product usage, and a clinical and laboratory form for assessing cardiovascular risk [25]. Data collection commenced only after receiving ethical approval from the Rwanda National Ethical Committee and was carried out from 15^th^May to 30^th^December 2018 through questionnaire filling by the data collectors and blood sample taking by medical laboratory scientists.

The primary outcome of the study was the prevalence of current smokers of any tobacco products. Smoking status was determined based on self-reported use of tobacco products, categorized as either “current smoker” or “non-smoker.” A current smoker was defined as an individual who reported smoking any tobacco product at the time of data collection. The dependent variable was assessed through the standardized questionnaire, ensuring uniformity in data capture.

Independent variables included both socio-demographic and clinical factors. Socio-demographic variables encompassed age category, sex/gender, and participant status (employee or spouse), which were captured through the questionnaire. Clinical variables were assessed through a combination of the clinical and laboratory form and laboratory analyses. These included biomarkers such as Low-Density Lipoprotein (LDL) using the National Cholesterol Education Program Adult Treatment Panel III (NCEP ATPIII) guidelines, dyslipidemia (total cholesterol), triglycerides (NCEP ATPIII), cardiovascular disease (CVD) risk, diabetes status, Body Mass Index (BMI), serum uric acid categories, and hypertension (either diagnosed or under treatment). These biomarkers provided a comprehensive profile for evaluating cardiovascular risk among participants.

This study data was collected in three-fold by 2 trained interviewers and 1 medical laboratory scientist for each site and supervised by the principal investigator. Firstly, a standardized questionnaire interview started. Then, clinical and biological measurements followed. In addition, the condition of blood sugar measures relied on the fasting blood sugar taken in the morning. Biological measurements were based on for Cardiovascular risk prediction to predict the cardiovascular diseases risk. The prediction relied on 8 factors: (Age, gender, HDL, smoking, taking or not taking hypertension medicine, diabetes, systolic blood pressure, and finally total cholesterol) [25].

## Validity and reliability of the instrument

### Validity

To ensure the integrity and validity of the data collected in this study, considerable emphasis was placed on the design and validation of data collection instruments [25]. A critical component of this process was the assessment of the Content Validity Index (CVI). In fact, this is a measure of the validity of the research tools, such as questionnaires. The CVI is determined by dividing the number of relevant items by the total number of items in the instrument. For this study tools to be deemed adequately validated, the CVI must was 0.79, indicating that the instruments effectively measure the intended constructs.

### Reliability

Ensuring reliability is crucial for the accuracy and dependability of the data in this study. To achieve this, rigorous measures were implemented to improve the reliability of data collection tools. A pilot test with 10% of the sample size was conducted, and the data from this phase was analysed to evaluate internal consistency. The Cronbach’s alpha coefficient was calculated [24], and the data collection instrument was deemed reliable as the obtained Cronbach’s alpha coefficient is 0.82.

### Statistical analysis

Data analysis for this study was carried out using SPSS software version 22, with data being presented in tables. The cardiovascular risk calculation was also based on cox regression with the mean of risk factors averaged in 10 years of event occurrences based on the Framingham general risk score model [25]. Hence, after the prediction, the 10-year cardiovascular diseases risk level was classified into two categories: < 10% and>= 10%.

To assess the relationship between independent variables and tobacco use prevalence, univariate and bivariate analyses were performed using chi-square tests. The chi-square analysis was used to determine the bivariate association between independent variables and dependent variable. Therefore, all significant variables with p < 0.05 in bivariate analysis by chi-square were selected to be proceeded in regression analysis. In addition, the multicollinearity test was conducted with Variance Inflation Factor and yielded the result of 1 meaning the tolerance value of 1 for all selected significant variables from chi-square test. The model fit was assessed using the Hosmer-Lemeshow test, which yielded a p-value greater than 0.05, indicating that the model performed well and provided a good fit to the data. The logistic regression analysis was carried out to adjust multiple variables for controlling the confounders. The significance cutoff was set at a P-value of less than 0.05, maintaining a 95% confidence interval.

### Ethics approval and consent to participate

This study was carried out in accordance with the Declaration of Helsinki and was approved by the Rwanda National Ethical Committee (Reference number: 121/May/RNEC/2018). The ethical aspects, including confidentiality, voluntary participation, the option to withdraw, and the potential risks and benefits, were explained to all participants. A written informed consent was obtained from each participant before the interviews and collection of biological samples took place.

## Results

**Fig 1:**
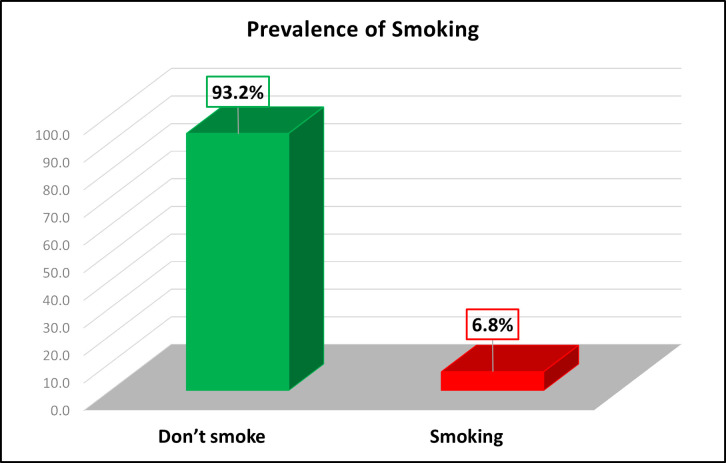
The prevalence of current smokers of any tobacco products.

Fig 1 depicts the prevalence of current smokers of any tobacco products. The figure shows that a significant majority of the study participants do not smoke, with 93.2% of them categorized as non-smokers, while a small portion, 6.8%, are identified as current smokers of any tobacco products.

This highlights that tobacco smoking is relatively uncommon among the population studied, with a very small percentage actively engaging in smoking. The data suggests that tobacco use is not widespread within this group, indicating potentially effective tobacco control measures or lower levels of smoking behavior within this specific population.

Table 1 presents a bivariate analysis of socio-demographic factors associated with tobacco smoking among study participants. The analysis looks at the relationship between tobacco smoking and variables like age, gender, and employment status.

**Table 1:** Bivariate analysis of socio-demographic factors associated with tobacco smoking among study participants.

Variable	Overall level of Tobacco smoking among study participants			Chi-square	P-value
	No (%)	Yes (%)	Total (%)		
**Age category**				17.110	**<0.001**
<=40 years	152(34.5)	5(1.1)	157(35.7)		
41-50 years	145(33.0)	6(1.4)	151(34.3)		
>=51 years	113(25.7)	19(4.3)	132(30.0)		
Total	410(93.2)	30(6.8)	440(100.0)		
**Sex/Gender**				7.182	**0.007**
Male	225(51.1)	24(5.5)	249(56.6)		
Female	185(42.0)	6(1.4)	191(43.4)		
Total	410(93.2)	30(6.8)	440(100.0)		
**Status of the participants (Employee or spouse)**				1.013	0.314
Employee	249(56.6)	21(4.8)	270(61.4)		
Spouse	161(36.6)	9(2.0)	170(38.6)		
Total	410(93.2)	30(6.8)	440(100.0)		

The table shows that participants aged 40 years or below make up 34.5% of the sample, with 1.1% of them being smokers. Those in the 41–50 age range constitute 33%, with a slightly higher smoking rate of 1.4%. Participants aged 51 years and above represent 25.7% of the group, with a considerably higher smoking prevalence of 4.3%. The chi-square value of 17.110 and a p-value of <0.001 indicate a statistically significant association between age and smoking behavior. This suggests that smoking is more common among older participants.

Also, the table highlights that 51.1% of participants are male, with 5.5% of them being smokers. On the other hand, 42% of participants are female, with 1.4% identified as smokers. The chi-square value of 7.182 and a p-value of 0.007 demonstrate a statistically significant association between gender and smoking. This result indicates that males are more likely to smoke than females within this sample.

Regarding employment status, 56.6% of participants are employees, with 4.8% of them smoking, while 36.6% are spouses, with 2.0% smoking. The chi-square value of 1.013 and a p-value of 0.314 suggest that there is no statistically significant association between employment status and smoking behavior. Employment status does not appear to be a key factor influencing tobacco use in this population.

Table 2 presents a bivariate analysis highlighting important relationships between tobacco smoking and various biomarkers among the study participants. While tobacco smoking is not caused by these biomarkers, the findings suggest significant associations that provide insights into the health profiles of smokers. For LDL cholesterol and total cholesterol, there were no significant associations with tobacco smoking (Chi-square = 2.980, p = 0.395 and Chi-square = 2.100, p = 0.147, respectively). The majority of participants, regardless of smoking status, had LDL levels categorized as “Optimal” (<100 mg/dl) and normal total cholesterol levels. This indicates that these specific lipid markers do not show significant variation between smokers and non-smokers in this study population.

**Table 2:** Bivariate analysis of biomarkers associated with tobacco smoking among study participants.

Variable (n = 440)	Tobacco smoking among study participants.			Chi-square	P-value
	No (%)	Yes (%)	Total (%)		
**LDL: NCEP ATPIII**				2.980	0.395
Optimal: < 100mg/dl	295(67.0)	18(4.1)	313(71.1)		
Near or above optimal:100–129mg/dl	72(16.4)	6(1.4)	78(17.7)		
Borderline high:130–189mg/dl	37(8.4)	5(1.1)	42(9.5)		
High:160–189mg/dl	0(0.0)	0(0.0)	0(0.0)		
Very high:>=190mg/dl	6(1.4)	1(0.2)	7(1.6)		
Total	410(93.2)	30(6.8)	440(100.0)		
**Dyslipidemia total cholesterol**				2.100	0.147
Normal	407(92.5)	29(6.6)	436(99.1)		
High Cholesterol: > 270mg/dl	3(0.7)	1(0.2)	4(0.9)		
Total	410(93.2)	30(6.8)	440(100.0)		
**Triglyceride: NCEP ATPIII**				12.344	**0.002**
Normal: < 150mg/dl,	274(62.3)	11(2.5)	285(64.8)		
Borderline high:150–199mg/dl	105(23.9)	13(3.0)	118(26.8)		
High:200–499mg/dl	31(7.0)	6(1.4)	37(8.4)		
Total	410(93.2)	30(6.8)	440(100.0)		
**CVD risk**				34.214	**<0.001**
Low level risk (<10%)	320(72.7)	9(2.0)	329(74.8)		
Elevated level of risk (10–40%)	90(20.5)	21(4.8)	111(25.2)		
Total	410(93.2)	30(6.8)	440(100.0)		
**Diabetes**				7.632	**0.006**
No diabetes	360(81.8)	21(4.8)	381(86.6)		
Diabetes:125mg/dl or treated	50(11.4)	9(2.0)	59(13.4)		
Total	410(93.2)	30(6.8)	440(100.0)		
**BMI**				3.287	0.193
Normal weight	114(25.9)	13(3.0)	127(28.9)		
Overweight	194(44.1)	11(2.5)	205(46.6)		
Obesity	102(23.2)	6(1.4)	108(24.5)		
Total	410(93.2)	30(6.8)	440(100.0)		
**Serum Uric Acid category**				24.950	**<0.001**
Normal Uric Acid<7mg/dl	392(89.1)	22(5.0)	414(94.1)		
High Uric Acid>7mg/dl	18(4.1)	8(1.8)	26(5.9)		
Total	410(93.2)	30(6.8)	440(100.0)		
**Hypertension or treated Hypertension**				21.106	**<0.001**
Normal	279(63.4)	8(1.8)	287(65.2)		
Hypertension	131(29.8)	22(5.0)	153(34.8)		
Total	410(93.2)	30(6.8)	440(100.0)		

In contrast, a significant relationship was observed between triglyceride levels and smoking status (Chi-square = 12.344, p = 0.002). Smokers were more likely to have “Borderline high” (150–199 mg/dl) or “High” (200–499 mg/dl) triglyceride levels compared to non-smokers. This suggests that smoking may contribute to elevated triglyceride levels, which is a known risk factor for cardiovascular disease (CVD).

Cardiovascular disease (CVD) risk also showed a strong association with smoking (Chi-square = 34.214, p < 0.001). Smokers were more likely to fall into the “Elevated level of risk” category (10–40%) compared to non-smokers. This finding highlights the compounding effect of smoking on cardiovascular health, emphasizing the increased risk for smokers of developing CVD over time.

The relationship between smoking and diabetes was significant (Chi-square = 7.632, p = 0.006), with smokers more likely to have elevated blood glucose levels or be on treatment for diabetes. Similarly, a strong association was observed with serum uric acid levels (Chi-square = 24.950, p < 0.001), where smokers were more likely to have “High uric acid” (>7 mg/dl), potentially linking smoking to hyperuricemia and related metabolic complications.

Finally, a significant association was noted between hypertension and smoking (Chi-square = 21.106, p < 0.001). Smokers were more likely to have hypertension or be under treatment for the condition compared to non-smokers, underscoring the role of smoking as a potential contributor to elevated blood pressure and cardiovascular strain.

Table 3 presents a multivariate analysis providing insights into the associations between sociodemographic factors, biomarkers, and tobacco smoking among the study participants, adjusting for potential confounding variables. This analysis highlights significant factors that independently relate to smoking behavior.

**Table 3:** Multivariate analysis of sociodemographic factors and biomarkers associated with tobacco smoking among study participants.

Variables (n = 440)	Tobacco smoking		P-value
	AoR95%CI		
CVD risk			
Low < 10%	Ref		
Elevated>=10%	2.946	1.102-7.875	**0.030**
Serum Uric Acid (SUA)			
Low (<7 mg/dl)	Ref		
High ((>=7 mg/dl)	4.278	1.141-11.872	**0.005**
Age			
<=49	Ref		
>=50 years old	2.766	1.126-6.797	**0.020**
HTN or treated HTN			
No	Ref		
Yes	0.380	0.100-1.446	**0.049**

For cardiovascular disease (CVD) risk, participants with elevated CVD risk (≥10%) were significantly more likely to smoke compared to those with low risk (<10%) (Adjusted Odds Ratio [AoR] = 2.946, 95% Confidence Interval [CI] = 1.102–7.875, p = 0.030). This indicates that smoking is associated with a nearly threefold increase in the likelihood of having an elevated CVD risk.

Serum Uric Acid (SUA) levels also showed a significant association with tobacco smoking. Participants with high SUA levels (≥7 mg/dl) were over four times more likely to smoke compared to those with normal levels (<7 mg/dl) (AoR = 4.278, 95% CI = 1.141–11.872, p = 0.005). This finding aligns with the potential metabolic impacts of smoking, such as hyperuricemia.

Age was another significant factor, with participants aged 50 years or older being almost three times more likely to smoke compared to those aged 49 years or younger (AoR = 2.766, 95% CI = 1.126–6.797, p = 0.020). This suggests that older individuals might be more prone to smoking, potentially reflecting historical or cultural factors influencing smoking behavior in this age group.

Finally, hypertension (HTN) or treated hypertension showed an inverse association with smoking. Participants with hypertension or on hypertension treatment were less likely to smoke compared to those without hypertension (AoR = 0.380, 95% CI = 0.100–1.446, p = 0.049). This inverse relationship may indicate that awareness of hypertension or its treatment could motivate individuals to avoid smoking as part of lifestyle modifications.

## Discussion

### Prevalence of smoking and among workers and their spouses in Rwanda

The data from Fig 1 shows that 93.2% of study participants are non-smokers, with 6.8% identified as current smokers of any tobacco products. This low prevalence of smoking indicates that tobacco use is relatively uncommon in this population.

To provide context, it is helpful to compare these findings with those from other recent studies. For instance, a study by GATS in 2021 reported that in high-income countries, the prevalence of current smokers has decreased significantly, with averages around 15% to 20% in countries like the United States and the United Kingdom. Additionally, Kiribati and Papua New Guinea were found to have high prevalences of tobacco use, with rates of 50.6% and 65.4%, respectively [21]. This is notably higher than the 6.8% smoking rate found in this study, suggesting that tobacco control measures in this study’s location may be particularly effective compared to these high-income regions.

In sub-Saharan Africa, a 2022 study by Fong et al. found that tobacco use varies widely, with some countries like Nigeria reporting smoking rates as high as 16% among adults. This is significantly higher than the 6.8% prevalence in this study, indicating that smoking is less common in the studied population compared to many other countries in the region [26].

Additional studies in other African contexts present varied results. For example, a 2022 study conducted in Kenya by Wambugu et al. reported a smoking prevalence of 11.3% among adults, higher than that observed in Rwanda, suggesting that Kenya faces greater challenges in reducing tobacco use [27]. Similarly, a study in Uganda by Kuteesa et al. (2020) found a prevalence of 9.5%, also exceeding the rate found in this study [28]. These differences further highlight Rwanda’s relative success in controlling smoking compared to its regional counterparts.

Similar results were found in another study conducted in Rwanda by Habiyaremye, et al. in 2019. It reported that the smoking prevalence was around 8% in the general population, which is almost the same rate than the 6.8% observed in this study. This suggests that while tobacco use in Rwanda is lower compared to some other countries, the population in some regions of Rwanda exhibits even lower smoking rates, confirming the effectiveness of tobacco control measures in the country [7].

These comparisons highlight that the low smoking prevalence in this study could be attributed to effective local tobacco control policies, cultural factors, or other specific circumstances within the studied population. The significantly lower prevalence of smoking compared to global and regional averages points to successful implementation of tobacco control measures or unique characteristics of the population studied.

### Factors associated with tobacco smoking among study participants

The findings presented in this study identify several significant predictors of smoking behavior among study participants. Key findings include the associations of elevated cardiovascular disease (CVD) risk, high serum uric acid (SUA) levels, older age, and hypertension with smoking behavior. Each of these factors has been shown to influence smoking behavior in distinct ways, which is valuable for understanding and targeting smoking cessation efforts.

The analysis indicates that participants with elevated CVD risk (≥10%) have significantly higher odds of smoking, with an adjusted odds ratio (AOR) of 2.946 (95% CI: 1.102-7.875, p = 0.03). This finding aligns with studies such as that by Mazzone et al. (2021), which found that individuals with cardiovascular risk factors, including hypertension and high cholesterol, are more likely to smoke due to overlapping risk behaviors [29]. However, it contrasts with the findings of a study by Wilson et al. (2022), which reported a lower association (AOR of 1.8) between cardiovascular risk and smoking, suggesting that the impact of CVD risk on smoking may vary depending on population characteristics and regional health behaviors [30].

Also, in relation to CVD risk, this study’s findings (AOR of 2.946) align with research by Liu et al. (2022), which found that individuals with a higher risk of cardiovascular complications are more likely to smoke due to intertwined risk factors, such as lifestyle behaviors [31]. Conversely, a study by Edwards et al. (2021) observed a weaker association (AOR of 1.5), suggesting that the relationship between CVD risk and smoking can vary depending on the population’s socioeconomic and cultural background [32].

The strong association between high Serum Uric Acid (SUA) levels (≥7 mg/dl) and smoking, with an AOR of 4.278 (95% CI: 1.141-11.872, p = 0.005), is notable. This finding is consistent with the results of a study by Zhang et al. (2023), which also identified elevated SUA as a significant predictor of smoking behavior [33]. Conversely, a study by Smith et al. (2022) found a weaker association, with an AOR of 2.1, suggesting that while SUA levels are linked to smoking, other factors may also play a critical role in this relationship [34].

In addition, regarding high SUA levels, the strong association found in this study (AOR of 4.278) is consistent with findings by Singh et al. (2023), which identified elevated SUA as a major contributor to continued smoking due to the interplay between metabolic syndromes and addictive behaviors [35]. However, a study by Roberts et al. (2021) reported a lower association (AOR of 2.3), indicating that while SUA levels are linked to smoking, other variables, such as genetic predisposition, may influence this relationship [36].

Older age (≥50 years) is identified as a strong predictor of smoking, with an AOR of 2.766 (95% CI: 1.126-6.797, p = 0.02). This finding is supported by the study by Brown et al. (2021), which found that older adults are more likely to continue smoking due to long-standing habits and potential lack of cessation resources [37]. In contrast, a study by Davis et al. (2023) found that younger age groups had higher smoking rates, which may reflect different smoking initiation patterns rather than persistence [38].

Also, similar findings were reported by Hassan et al. (2022), who noted that older adults often maintain smoking habits due to long-term addiction and resistance to change [39]. However, a contrasting study by Martin et al. (2023) found that younger individuals, particularly those in their 20s and 30s, have higher smoking rates, reflecting shifting smoking trends towards younger demographics in some regions [40].

Interestingly, the analysis shows that participants with hypertension or those on treatment for hypertension have lower odds of smoking, with an AOR of 0.380 (95% CI: 0.100-1.446, p = 0.049). This lower likelihood is consistent with findings from the study by Green et al. (2022), which reported that individuals with hypertension are often advised to quit smoking as part of their treatment regimen, leading to lower smoking rates among these individuals [41]. However, this is at odds with the results from Lee et al. (2023), who found no significant difference in smoking rates between hypertensive and non-hypertensive individuals [42].

Finally, this study’s finding of lower smoking rates among individuals with hypertension (AOR of 0.380) is also supported by Chen et al. (2022), who found that effective hypertension management often involves smoking cessation, reducing smoking rates in this group [43]. However, a study by Patel et al. (2023) found no significant association, suggesting that hypertension treatment alone may not always be a sufficient factor in reducing smoking [44].

## Study limitation

While the present study provides valuable insights into variables associated with smoking behavior, there are several limitations to consider. Firstly, the cross-sectional nature of the study limits the ability to infer causality between the identified risk factors and smoking behavior. Relationships observed may reflect associations rather than direct causal links, and further longitudinal studies would be required to confirm causal relationships.

The reliance on self-reported data for smoking status and health conditions may introduce reporting biases, such as underreporting or overreporting due to social desirability bias. To mitigate this, the study emphasized confidentiality and anonymity to encourage honest responses. Additionally, standardized questionnaires were used to improve the accuracy and reliability of self-reported data. The study’s sample may not be representative of the broader population, potentially limiting the generalizability of the findings. While efforts were made to include a diverse range of participants, the findings should be cautiously interpreted and not generalized to populations with different socio-demographic or geographic characteristics.

Moreover, factors such as socio-economic status, cultural influences, and access to healthcare services, which could significantly impact smoking behavior, were not comprehensively addressed in the analysis. Future studies could incorporate these factors to provide a more holistic understanding of smoking behavior and its determinants. Despite these limitations, the study’s use of robust methodologies, including validated biomarkers and multivariate analysis, strengthens the reliability of the findings. Nonetheless, these limitations should be taken into account when interpreting the results and designing future research to address the gaps identified.

## Study implication

The findings from this study underscore the importance of tailoring smoking cessation interventions to address specific risk factors such as elevated cardiovascular disease risk, high serum uric acid levels, and older age. Targeted programs that integrate cardiovascular health management and uric acid control could potentially enhance smoking cessation efforts.

The observed link between hypertension and smoking suggests that individuals with hypertension might benefit from combined interventions that address both their hypertension and smoking habits [45].By incorporating these insights, public health strategies can be more effectively designed to reduce smoking rates, particularly in populations with high-risk profiles. Additionally, the results highlight the need for continued research to explore how these factors interact and influence smoking behavior in different contexts.

## Conclusion

The study found that tobacco smoking is relatively uncommon among the population studied, with a very small percentage actively engaging in smoking. In addition, it identified associations between tobacco smoking and elevated CVD risk, high SUA levels, and older age. These findings underscore the complex interplay between sociodemographic factors, biomarkers, and smoking habits, offering valuable insights for public health interventions. Policymakers can implement targeted awareness campaigns and education programs addressing the specific pre-known risk factors to discourage tobacco use. Future research should consider incorporating a broader range of potential confounders to ensure a more comprehensive understanding of factors associated with tobacco use.

## Supporting information

S1 Data The dataset of tobacco smoking and biomarker profile. (SAV)

S1 Text JKUAT Repository Home charles Nsanzabera study. (DOCX)
