# Supplementary material for: Tobacco smoking and biomarker profile among beverage industrial workers and their spouses in Rwanda: A cross-sectional study
Source: PLOS Glob Public Health. 2025 Jul 3;5(7):e0003946. doi: 10.1371/journal.pgph.0003946 (PMC12225804; doi:10.1371/journal.pgph.0003946)
Supplement: S1 Text — (DOCX) [file pgph.0003946.s002.docx]

**JKUAT Repository Home (For more information)**

http://ir.jkuat.ac.ke/bitstream/handle/123456789/5895/Nsanzabera%2C%20Charles%20PhD%20Public%20Health%2C%202022%287%29%20%284%29.pdf?sequence=1&isAllowed=y
